# Assessment of a Hotel-Based COVID-19 Isolation and Quarantine Strategy for Persons Experiencing Homelessness

**DOI:** 10.1001/jamanetworkopen.2021.0490

**Published:** 2021-03-02

**Authors:** Jonathan D. Fuchs, Henry Clay Carter, Jennifer Evans, Dave Graham-Squire, Elizabeth Imbert, Jessica Bloome, Charles Fann, Tobi Skotnes, Jonathan Sears, Rebecca Pfeifer-Rosenblum, Alice Moughamian, Joanna Eveland, Amber Reed, Deborah Borne, Michele Lee, Molly Rosenthal, Vivek Jain, Naveena Bobba, Margot Kushel, Hemal K. Kanzaria

**Affiliations:** 1 San Francisco Department of Public Health, San Francisco, California; 2 Department of Medicine, University of California, San Francisco; 3 Benioff Homelessness and Housing Initiative, University of California, San Francisco; 4 Division of HIV, Infectious Diseases and Global Medicine, Zuckerberg San Francisco General Hospital, University of California, San Francisco; 5 Department of Emergency Medicine, University of California, San Francisco

## Abstract

**Importance:**

Several jurisdictions in the United States have secured hotels to temporarily house people experiencing homelessness who require isolation or quarantine for confirmed or suspected coronavirus disease 2019 (COVID-19). To our knowledge, little is known about how these programs serve this vulnerable population outside the hospital setting.

**Objective:**

To assess the safety of a hotel-based isolation and quarantine (I/Q) care system and its association with inpatient hospital capacity.

**Design, Setting, and Participants:**

This retrospective cohort study of a hotel-based I/Q care system for homeless and unstably housed individuals in San Francisco, California, was conducted from March 19 to May 31, 2020. Individuals unable to safely isolate or quarantine at home with mild to moderate COVID-19, persons under investigation, or close contacts were referred from hospitals, outpatient settings, and public health surveillance to 5 I/Q hotels. Of 1009 I/Q hotel guests, 346 were transferred from a large county public hospital serving patients experiencing homelessness.

**Exposure:**

A physician-supervised team of nurses and health workers provided around-the-clock support, including symptom monitoring, wellness checks, meals, harm-reduction services, and medications for opioid use disorder.

**Main Outcomes and Measures:**

Characteristics of I/Q hotel guests, program retention, county hospital readmissions, and mean length of stay.

**Results:**

Overall, the 1009 I/Q hotel guests had a median age of 44 years (interquartile range, 33-55 years), 756 (75%) were men, 454 (45%) were Latinx, and 501 (50%) were persons experiencing sheltered (n = 295) or unsheltered (n = 206) homelessness. Overall, 463 (46%) received a diagnosis of COVID-19; 303 of 907 (33%) had comorbid medical disorders, 225 of 907 (25%) had comorbid mental health disorders, and 236 of 907 (26%) had comorbid substance use disorders. A total of 776 of 955 guests (81%) completed their I/Q hotel stay; factors most strongly associated with premature discontinuation were unsheltered homelessness (adjusted odds ratio, 4.5; 95% CI, 2.3-8.6; *P* < .001) and quarantine status (adjusted odds ratio, 2.6; 95% CI, 1.5-4.6; *P* = .001). In total, 346 of 549 patients (63%) were transferred from the county hospital; of 113 ineligible referrals, 48 patients (42%) had behavioral health needs exceeding I/Q hotel capabilities. Thirteen of the 346 patients transferred from the county hospital (4%) were readmitted for worsening COVID-19. Overall, direct transfers to I/Q hotels from emergency and outpatient departments were associated with averting many hospital admissions. There was a nonsignificant decrease in the mean hospital length of stay for inpatients with confirmed or suspected COVID-19 from 5.5 to 2.7 days from March to May 2020 (*P* = .11).

**Conclusions and Relevance:**

To support persons experiencing homelessness during the COVID-19 pandemic, San Francisco rapidly and safely scaled a hotel-based model of I/Q that was associated with reduced strain on inpatient capacity. Strategies to improve guest retention and address behavioral health needs not met in hotel settings are intervention priorities.

## Key Points

**Question:**

Can persons experiencing homelessness with confirmed or suspected coronavirus disease 2019 (COVID-19) and mild to moderate symptoms be safely monitored in designated isolation and quarantine (I/Q) hotels?

**Findings:**

In this cohort study among 1009 I/Q hotel guests referred from hospitals, outpatient settings, and public health surveillance, 81% completed their recommended I/Q course, and only 4% of those transferred from the county hospital required readmission for COVID-19 progression.

**Meaning:**

This study suggests that, during the COVID-19 pandemic, a hotel-based I/Q strategy that delivers integrated medical and behavioral health support to people experiencing homelessness can be done safely outside the hospital setting.

## Teaser

This cohort study assesses the safety of a hotel-based isolation and quarantine care system and its association with hospital inpatient capacity during the COVID-19 pandemic.

## Introduction

Isolation of individuals with severe acute respiratory syndrome coronavirus 2 (SARS-CoV-2) infection and quarantine of close contacts are key public health interventions to limit the population-level spread of infection.^1,2,3^ However, individuals with coronavirus disease 2019 (COVID-19) who are homeless, unstably housed, or living in congregate settings or dense households face key structural barriers to isolation.^4^ With an estimated 568 000 people experiencing homelessness each night in the United States^5^ and numerous outbreaks of COVID-19 in homeless shelters,^6,7^ there is a pressing need for noncongregate solutions to support isolation of individuals in this population with COVID-19.

Since the COVID-19 pandemic began, several jurisdictions have used private hotels to secure voluntary, temporary housing for individuals with suspected or confirmed COVID-19 who are recovering from mild to moderate disease.^8,9^ Without this option, persons experiencing homelessness and requiring isolation might need prolonged hospitalization while they are infectious, straining valuable hospital capacity. Given the excess mortality in hospitals overwhelmed by admissions of patients with COVID-19,^10,11^ maintaining alternative housing to meet the needs of patients with SARS-CoV-2 infection is a priority for safety-net hospitals caring for large numbers of homeless individuals.

On March 19, 2020, 2 weeks after the first identified cases of COVID-19 in San Francisco, California, and 3 days after the city declared one of the nation’s first shelter-in-place health orders,^12^ we accepted patients as guests into the first of 5 isolation and quarantine (I/Q) hotels that delivered integrated medical and behavioral health services to homeless and other marginally housed persons. Here, we describe the populations served by the I/Q hotels and examine factors associated with individuals leaving I/Q hotels earlier than recommended (which could fuel community transmission).^13^ Finally, we explore how the availability of I/Q hotels was associated with hospital capacity at our public county hospital, Zuckerberg San Francisco General (ZSFG), where homeless individuals account for one-third of annual admissions.

## Methods

### Study Design and Setting

We conducted a retrospective cohort study of persons with confirmed or suspected COVID-19 who were eligible for temporary stays in 5 designated I/Q hotels with 457 beds, leased by the City and County of San Francisco under its alternative housing program.^14^ The I/Q hotels were centrally located near several homeless shelters and were the only alternative care site for individuals with confirmed or suspected COVID-19 who were experiencing homelessness or unstable housing or living in dense congregate settings. This analysis focuses on individuals transferred to I/Q hotels from San Francisco hospitals, homeless shelters, single-room occupancy hotels, and other community sites from March 19 to May 31, 2020. We further characterized a subset of patients transferred from ZSFG inpatient wards, the emergency department, urgent care, and 4 ambulatory care clinics on the hospital campus. We report results in accordance with the Strengthening the Reporting of Observational Studies in Epidemiology (STROBE) reporting guideline. The University of California, San Francisco institutional review board approved this public health program evaluation and granted waivers for individual informed consent for public health evaluation programs under 45 CFR 46.11(c).

### Participants and Program Description

**Figure 1.**
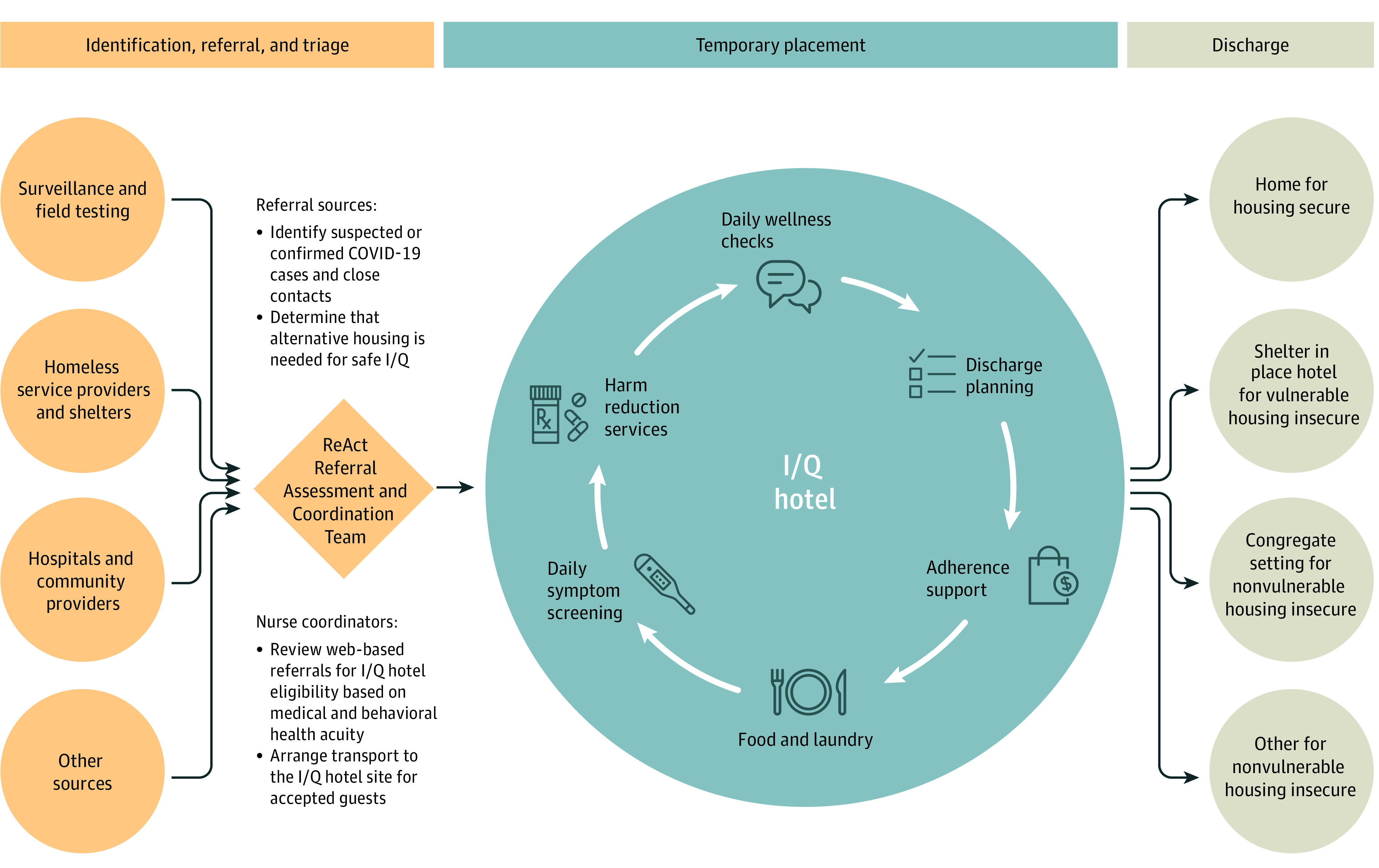
Isolation and Quarantine (I/Q) Hotel System of Care COVID-19 indicates coronavirus disease 2019.

A physician-supervised team of nurses, health workers, and security staff provided free, around-the-clock support to hotel guests who had COVID-19, were persons under investigation, or were close contacts with known SARS-CoV-2 exposures (Figure 1). A total of 1 to 3 nurses oversaw the care of 50 to 150 guests with ratios accommodating patient acuity and volume of intakes. We followed the Centers for Disease Control and Prevention guidelines to define the isolation period for those with symptomatic and asymptomatic COVID-19 and the duration of quarantine among close contacts.^15,16^ Nurses assessed patients for I/Q hotel eligibility using a web-based screening form (eMethods in the Supplement). Criteria included self-reported inability to isolate (eg, sharing a tent) and inhabiting a shelter or shared living space where physical distancing more than 6 feet (1.8 m) from others or disinfection of shared spaces was not possible. Patients were ineligible if they required medical or behavioral health support beyond what could be provided in the hotel (eg, severe symptoms requiring regular medical intervention^17^), required help with activities of daily living or taking prescribed medications, experienced recent alcohol withdrawal seizures, or were unable to self-regulate behaviors that would make isolation challenging.

Guests were monitored for symptoms by on-site nursing staff and received twice daily telephone call wellness checks, meals that accommodated dietary restrictions, and hygiene kits. Those whose alcohol or other substance use created a barrier to adherence or a safety risk were offered addiction medicine physician consultation via telemedicine.^18,19^ Addiction treatment included evidence-based pharmacotherapy for opioid use disorders and counseling for stimulant use disorders. We offered an array of harm-reduction services, including access to safe consumption supplies and designated smoking areas, intranasal naloxone, nicotine replacement, medical cannabis, and a managed alcohol program. To prevent alcohol withdrawal, we supplied donated alcohol (beer or vodka), dosed twice daily to a maximum of 10 standard drink equivalents per day. Additional hotel retention strategies included temporary storage for guests’ belongings; laundry services; accommodations for children, including diapers and formula; the ability to support pets on-site; twice daily telephone calls from I/Q adherence counselors; and $20 gift cards after completing their stay in the I/Q hotel. On discharge from the I/Q hotel, guests returned home or, if they were homeless, to a shelter-in-place hotel if they were at risk for severe COVID-19 disease or to a congregate shelter if they were not.^20^

### Data Sources

We used 4 administrative and clinical data sources to conduct this study. We extracted referral sources and demographic and clinical data, including reasons for leaving the I/Q hotel early, from a web-based intake and bed management system, GetCare (RTZ Systems Inc). The ZSFG Department of Care Coordination created an Excel-based system (Microsoft Corp) for I/Q hotel referrals to track a patient’s name, medical record number, date of birth, ZSFG campus location, date of referral, date of transfer, and reason for denial, if applicable. The hospital’s electronic health record (Epic) was reviewed for SARS-CoV-2 test results of referred patients, length of stay for inpatients, and clinical presentation and mortality for any patients sent back to the hospital from an I/Q hotel. Finally, we linked records from GetCare to the county’s Coordinated Care Management System (CCMS), an integrated, interagency clinical and social service delivery data set. A detailed description of the CCMS and the definitions of study measures used from the data set can be found elsewhere.^21^

### Measures

To describe the characteristics of individuals accessing the I/Q program, study measures included age, gender, race/ethnicity, history of homelessness, length of time homeless, current housing status, recent jail health encounter, source of referral, and COVID-19 status as measured by results of SARS-CoV-2 real-time reverse transcription polymerase chain reaction test. For individuals with CCMS-linked records, we assessed a history of Elixhauser medical conditions, mental health disorders, and substance use disorders that are associated with early mortality.^22^

To assess program retention, we included all guests with available I/Q hotel discharge information. We compared discharges prior to the recommended I/Q course^15,16^ with discharges after a complete I/Q stay. We defined premature discontinuation of I/Q as leaving voluntarily prior to the end of the prescribed isolation period, either against medical advice or declining I/Q stay after arrival. We did not include in this definition those who were transferred to a health care facility for a higher level of care, discharged for administrative reasons (eg, a room could not accommodate a guest with physical disabilities), or asked to leave to protect staff in the face of unsafe guest behavior. We assessed the association between premature discontinuation and demographic and observable risk factors.

To explore the association of the I/Q hotel program with hospital capacity, we assessed patients transferred from ZSFG, comparing the number and percentage of referrals from preinpatient and inpatient settings. We ascertained the number of successful transfers to I/Q hotels, the reasons for ineligibility, hospital length of stay, and hospital readmissions.

### Statistical Analysis

We assessed sample characteristics via measures of central tendency and statistical dispersion. We used χ^2^ tests to compare frequencies of exposure variables by I/Q completion status, and we used logistic regression with bivariate odds ratios (ORs) to evaluate the association between exposure variables and premature discontinuation of I/Q. We used multivariable logistic regression to examine the independent associations of exposure variables and premature discontinuation of I/Q, adjusting for hypothesized confounders (age, gender, and race/ethnicity) and month of referral. We report adjusted ORs (AORs) and 95% CIs. We conducted all analyses using 2-sided tests, with *P* < .05 considered statistically significant, and considered interactions between significant variables. We used Stata, version 16 (StataCorp) for all analyses.

## Results

Overall, 1065 accepted referrals resulted in 1009 unique individuals entering I/Q hotels. In total, (1009) 95% had 1 placement, (51) 5% had 2 placements, and (5) less than 1% had 3 placements. The median hotel length of stay was 10 days (interquartile range, 5-16 days). As seen in Table 1, 756 guests (75%) were male, the median age was 44 years (interquartile range, 33-55 years), and 454 (45%) were Latinx. A total of 501 guests (50%) were either sheltered or unsheltered homeless, and more than one-third (367 [36%]) were referred to an I/Q hotel from a hospital. At the time of hotel entry, 463 guests (46%) had a laboratory-confirmed COVID-19 diagnosis, 379 (38%) were persons under investigation awaiting test results, and 146 (15%) were close contacts to a person known to have COVID-19 requiring quarantine. Of the sample, 90% (n = 907) had matched CCMS records; 303 of these patients (33%) had an Elixhauser medical condition, 225 (25%) had Elixhauser mental health disorders, and 236 (26%) had Elixhauser substance use disorders. In the past year, 91 individuals (10%) had a jail health encounter. There were no differences in COVID-19 status or premature discontinuation of I/Q among those with or without CCMS-matched records. However, CCMS-matched individuals were more likely than unmatched individuals to be older, male, homeless, and White (eTable 1 in the Supplement).

**Table 1.** Characteristics of Individuals Admitted to Isolation and Quarantine Hotels, March 19 to May 31, 2020

Characteristic	All placements, No. (%) (N = 1009)
Age, y	
<40	396 (39)
40-49	249 (25)
50-59	216 (21)
≥60	148 (15)
Gender	
Male	756 (75)
Female	239 (24)
Transgender	10 (1)
Other or unknown	4 (0.4)
Race/ethnicity	
White	235 (23)
Black	187 (19)
Latinx	454 (45)
Asian or Pacific Islander	78 (8)
Native American	13 (1)
Multiethnic	21 (2)
Refused or unknown	21 (2)
Living situation	
Home, apartment, RV, or trailer	282 (28)
Homeless	
Sheltered	295 (29)
Unsheltered	206 (20)
Single-room occupancy hotel	133 (13)
Congregate living setting	12 (1)
Other or unknown	81 (8)
Ever homeless (n = 907)^a^	
No	310 (34)
Yes	597 (66)
Referral source	
Outpatient	101 (10)
Hospital	367 (36)
Homeless service provider or shelter	169 (17)
Surveillance or field testing	106 (11)
Other	153 (15)
Missing	113 (11)
COVID-19 status	
COVID-19 diagnosis	463 (46)
PUI	379 (38)
Close contact	146 (15)
None of the above	5 (0.5)
Missing	16 (2)
Elixhauser medical condition (n = 907)^a^^,^^b^	
No	604 (67)
Yes	303 (33)
Elixhauser mental health disorder (n = 907)^a^^,^^c^	
No	682 (75)
Yes	225 (25)
Elixhauser substance use disorder (n = 907)^a^^,^^d^	
No	671 (74)
Yes	236 (26)
Jail stay in past year (n = 907)^a^	
No	816 (90)
Yes	91 (10)

Abbreviations: COVID-19, coronavirus disease 2019; PUI, persons under investigation; RV, recreational vehicle.

^a^ A total of 102 records were excluded because they were unmatched with administrative data. Homelessness in the Coordinated Care Management System (CCMS) is determined by a combination of 8 data fields that combine observed homeless service use (eg, shelter stay or homeless outreach team encounter) and self-reported homelessness (as reported by a patient during a health care encounter). Jail stay in the past year was derived from jail health encounter records in the CCMS. Jail health encounters are recorded in the CCMS as county jail detention, which requires a jail health evaluation every day of incarceration, and jail health records within the CCMS provide the jail length of stay.

^b^ Elixhauser medical condition was defined as having 2 or more diagnosis codes in the current and past 2 fiscal years in medical records and included rheumatic arthritis, neurologic disorders, paralysis, cancer, kidney failure, liver disease, peptic ulcer disease, hypothyroidism, weight loss, obesity, diabetes, fluid and electrolyte disorders, chronic pulmonary disease, pulmonary circulation disorder, peripheral vascular disease, hypertension, congestive heart failure, valvular disease, cardiac arrhythmias, coagulopathy, blood loss anemia, deficiency anemia, and HIV or AIDS.

^c^ Elixhauser mental health disorder was defined as having 2 or more diagnosis codes in the current and past 2 fiscal years in medical records and included psychoses and depression.

^d^ Elixhauser substance use disorder was defined as having 2 or more diagnosis codes in the current and past 2 fiscal years in medical records and included alcohol or drug use.

### Program Retention and Premature Discontinuation of I/Q

We included a total of 955 of 1009 guests (95%) in the analysis of retention and voluntary premature discontinuation of I/Q; 54 guests were excluded because of the following discharge reasons: needed a higher level of care (n = 40), administrative reasons (n = 6), and safety or other reasons (n = 8). In total, 776 of 955 guests (81%) completed their I/Q stay. Guests who completed I/Q stayed for a mean of 13.1 (9.2) days compared with 5.5 (6.0) days among guests who left prematurely.

In multivariable regression models, premature discontinuation of I/Q was strongly associated with unsheltered homeless status on admission to the hotel (AOR, 4.5; 95% CI, 2.3-8.6; *P* < .001) and requiring quarantine as a close contact (AOR, 2.6; 95% CI, 1.5-4.6; *P* = .001) (Table 2). Age younger than 40 years (AOR, 2.5; 95% CI, 1.3-4.8; *P* = .01), female gender (AOR, 1.8; 95% CI, 1.2-2.7; *P* = .01), Black or African American identification (AOR, 1.7; 95% CI, 1.0-2.9; *P* = .045), and referral later during the study period (AOR, 1.1; 95% CI, 1.0-1.2; *P* = .02) also were associated with premature discontinuation of I/Q. After adjusting for other covariates, Elixhauser medical condition, mental health disorder, substance use disorder, and having a jail health encounter in the past year were not associated with premature discontinuation. Interaction effects examining race/ethnicity and housing status were not significant (eTable 2 in the Supplement).

**Table 2.** Factors Associated With Premature Discontinuation of I/Q

Factor	Premature discontinuation, No./total No. (%)^a^	OR (95% CI)	*P* value	AOR (95% CI)^b^	*P* value
Age, y					
≥60	17/136 (12.5)	1 [Reference]	NA	1 [Reference]	NA
50-59	39/200 (19.5)	1.7 (0.9-3.1)	.09	1.9 (0.9-3.6)	.07
40-49	42/234 (18.0)	1.5 (0.8-2.8)	.17	1.8 (0.9-3.5)	.09
<40	81/385 (21.0)	1.9 (1.1-3.3)	.03	2.5 (1.3-4.8)	.01
Gender					
Male	121/721 (16.8)	1 [Reference]	NA	1 [Reference]	NA
Female	56/220 (25.5)	1.7 (1.1-2.4)	.004	1.8 (1.2-2.7)	.01
Transgender or other	2/14 (14.3)	0.8 (0.2-3.7)	.81	0.4 (0.1-2.0)	.26
Race/ethnicity					
White	43/217 (19.8)	1 [Reference]	NA	1 [Reference]	NA
Black	52/178 (29.2)	1.7 (1.0-2.7)	.03	1.7 (1.0-2.9)	.045
Latinx	63/432 (14.6)	0.7 (0.5-1.1)	.09	0.9 (0.5-1.5)	.94
Asian or Pacific Islander	8/76 (10.5)	0.5 (0.2-1.1)	.07	0.7 (0.3-1.6)	.36
Multiethnic, other, or unknown	13/52 (25.0)	1.3 (0.7-2.7)	.41	1.4 (0.6-3.2)	.39
Living situation					
Home, apartment, RV, or trailer	30/270 (11.1)	1 [Reference]	NA	1 [Reference]	NA
Homeless					
Sheltered	43/276 (15.6)	1.5 (0.9-2.4)	.13	1.7 (0.9-3.3)	.13
Unsheltered	76/192 (39.6)	5.2 (3.3-8.4)	<.001	4.5 (2.3-8.6)	<.001
Congregate living setting or SRO	17/140 (12.1)	1.1 (0.6-2.1)	.76	1.3 (0.7-2.7)	.42
Other or unknown	13/77 (16.9)	1.6 (0.8-3.3)	.18	1.2 (0.5-2.9)	.68
Referral source					
Outpatient	21/96 (21.9)	1 [Reference]	NA	1 [Reference]	NA
Hospital	84/341 (24.6)	1.2 (0.7-2.1)	.58	1.1 (0.6-2.0)	.75
Homeless service provider or shelter	23/158 (14.6)	0.6 (0.3-1.1)	.14	0.5 (0.3-1.1)	.10
Surveillance or field testing	12/106 (11.3)	0.5 (0.2-1.0)	.05	0.7 (0.3-1.7)	.48
Other	24/146 (16.4)	0.7 (0.4-1.3)	.29	0.6 (0.3-1.2)	.13
Missing	15/108 (13.9)	0.6 (0.3-1.2)	.14	0.5 (0.2-1.1)	.09
COVID-19 status					
COVID-19 diagnosis	47/438 (10.7)	1 [Reference]	NA	1 [Reference]	NA
PUI	89/358 (24.9)	2.8 (1.9-4.0)	<.001	1.5 (0.9-2.5)	.09
Close contact	37/141 (26.2)	3.0 (1.8-4.8)	<.001	2.6 (1.5-4.6)	.001
None of the above	2/5 (40.0)	5.5 (0.9-34.0)	.06	4.0 (0.6-29.0)	.17
Missing	4/13 (30.8)	3.7 (1.1-12.5)	.04	4.8 (1.1-21.7)	.04
Elixhauser medical condition^c^^,^^d^					
No	97/583 (16.6)	1 [Reference]	NA	1 [Reference]	NA
Yes	63/274 (23.0)	1.5 (1.0-2.1)	.03	1.4 (0.9-2.2)	.17
Elixhauser mental health disorder^d^^,^^e^					
No	110/654 (16.8)	1 [Reference]	NA	1 [Reference]	NA
Yes	50/203 (24.6)	1.6 (1.1-2.4)	.01	1.0 (0.6-1.6)	.89
Elixhauser substance use disorder^d^^,^^f^					
No	104/639 (16.3)	1 [Reference]	NA	1 [Reference]	NA
Yes	56/218 (25.7)	1.8 (1.2-2.6)	.002	0.8 (0.05-1.4)	.42
Jail stay in past year^d^					
No	131/772 (17.0)	1 [Reference]	NA	1 [Reference]	NA
Yes	29/85 (34.1)	2.5 (1.6-4.1)	<.001	1.6 (0.7-2.3)	.39

Abbreviations: AOR, adjusted odds ratio; COVID-19, coronavirus disease 2019; I/Q, isolation and quarantine; NA, not applicable; OR, odds ratio; PUI, persons under investigation; RV, recreational vehicle; SRO, single-room occupancy hotel.

^a^ N = 955 (excludes patients elevated to a higher level of care and discharges for administrative and safety reasons).

^b^ Model adjusted for calendar week of referral.

^c^ Elixhauser medical condition was defined as having 2 or more diagnosis codes in the current and past 2 fiscal years in medical records and included rheumatic arthritis, neurologic disorders, paralysis, cancer, kidney failure, liver disease, peptic ulcer disease, hypothyroidism, weight loss, obesity, diabetes, fluid and electrolyte disorders, chronic pulmonary disease, pulmonary circulation disorder, peripheral vascular disease, hypertension, congestive heart failure, valvular disease, cardiac arrhythmias, coagulopathy, blood loss anemia, deficiency anemia, and AIDS or HIV.

^d^ Data not shown for records unmatched to administrative data.

^e^ Elixhauser mental health disorder was defined as having 2 or more diagnosis codes in the current and past 2 fiscal years in medical records and included psychoses and depression.

^f^ Elixhauser substance use disorder was defined as having 2 or more diagnosis codes in the current and past 2 fiscal years in medical records and included alcohol or drug use.

We performed a sensitivity analysis among guests included in the regression analysis with available CCMS data (n = 857) (eTable 3 in the Supplement). Regression models adjusting for the length of time as homeless demonstrated only slight differences compared with the aforementioned model. Younger age, female gender, those with unsheltered housing status, close contacts, and referral later during the study period were independently associated with premature discontinuation. The magnitude of the association between Black or African American race/ethnicity and premature discontinuation remained unchanged but no longer reached statistical significance (AOR, 1.7; 95% CI, 1.0-2.9; *P* = .07).

### Transfers From ZSFG to I/Q Hotels

**Figure 2.**
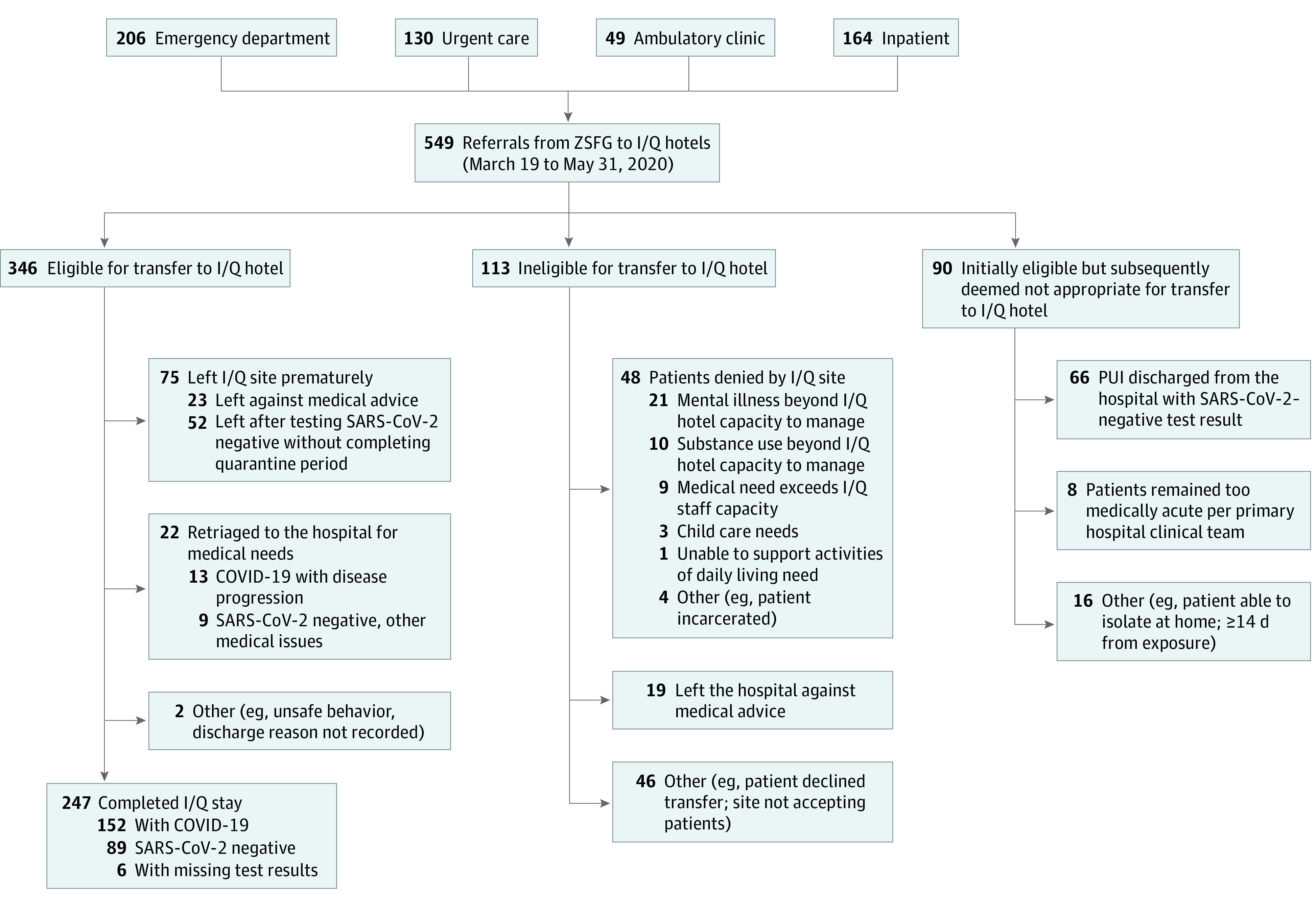
Flow Diagram of Referrals From Zuckerberg San Francisco General (ZSFG) Hospital Campus to Isolation and Quarantine (I/Q) Hotels, March 19 to May 31, 2020 The number of referrals (n = 346) exceeded the number of individuals transferred (n = 327) as individuals could be referred more than once. COVID-19 indicates coronavirus disease 2019; PUI, persons under investigation; and SARS-CoV-2, severe acute respiratory syndrome coronavirus 2.

During the 10-week study, ZSFG made 549 referrals to the I/Q hotel system. Of these, there were 346 (63%) successful transfers to I/Q hotels, representing 327 unique individuals. Overall, 308 individuals were referred once, while 19 had 2 or more referrals (Figure 2). Of these 327 individuals, 247 (76%) completed their I/Q hotel stay, most of whom (152 [62%]) had laboratory-confirmed COVID-19. Only 13 of 327 (4%) returned to the hospital for reassessment of suspected COVID-19 progression, of whom 1 died and 9 others required hospitalization for other medical and behavioral health conditions. No guests died during a stay at an I/Q hotel.

Of 549 referrals from ZSFG, 113 (21%) were ineligible (Figure 2). Of these, 48 (42%) had mental health needs or substance use disorders that exceeded I/Q hotel capabilities. A total of 90 other individuals referred from ZSFG were found to have a negative SARS-CoV-2 test result prior to hospital discharge or an alternative location for isolation having been found.

**Figure 3.**
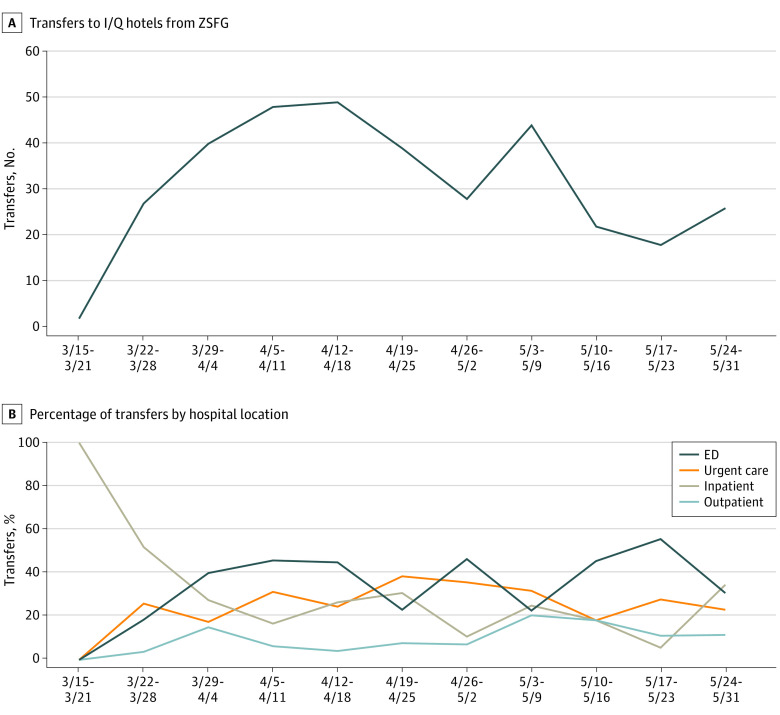
Total Isolation and Quarantine (I/Q) Hotel Transfers From Zuckerberg San Francisco General (ZSFG) Hospital by Hospital Referral Location, March 19 to May 31, 2020 (N = 346) A, Total number of patient transfers per week to I/Q hotels from ZSFG between March 19 and May 31, 2020. B, Percentage of transfers over time by hospital campus location (ie, emergency department [ED], urgent care, ambulatory care clinic, and inpatient unit). Overall, the proportion of referrals from inpatient units decreased over time, while referrals from other locations increased.

Figure 3 shows the number of transfers to I/Q hotels per week (Figure 3A) and by ZSFG location (Figure 3B). Most early transfers originated from the inpatient setting. Over time, an increasing proportion of transfers came from the emergency department, urgent care, and ambulatory care clinics, which averted the need for hospitalization altogether. In the last month of the study, 77% of referrals (85 of 110) were initiated in preinpatient hospital settings. The total number of successful I/Q hotel transfers, including those with COVID-19 and persons under investigation (n = 346), exceeded the number of all COVID-19 admissions to the hospital (n = 212) during the study period. We observed a nonstatistically significant reduction in the length of stay for inpatients with confirmed or suspected COVID-19. The mean number of inpatient days was 3.9 overall (n = 61), decreasing from 5.5 days in March to 3.8 days in April and to 2.7 days in May (1-way analysis of variance; *P* = .11).

## Discussion

With more than 8000 persons experiencing homelessness in San Francisco nightly^23^ and 18 000 low-income persons living in single-room occupancy hotels with shared kitchens and bathrooms,^24^ there was a critical need to establish an alternative housing program to contain the spread of SARS-CoV-2 in these vulnerable communities while preventing hospitals from becoming proxy I/Q facilities. During the first 10 weeks of operation (from March to May 2020), we established an I/Q hotel system of care that accommodated more than 1000 persons with suspected or confirmed COVID-19 despite reluctance from several hotel owners to offer temporary housing to people with COVID-19 and those experiencing homelessness.

Among guests with COVID-19, only 4% required hospital readmission owing to disease progression, a rate similar to that seen in other jurisdictions with similar programs.^8^ In a subsample referred from the city’s safety-net hospital, the number of successful transfers to I/Q hotels far exceeded the number of COVID-19–related admissions. In addition, direct transfers to I/Q hotels from preinpatient locations increased, which was a factor associated with averting hospitalizations entirely. Concomitantly, we observed a decrease in hospital length of stay for inpatients with COVID-19 from 5.5 days to 2.7 days. The I/Q system may have helped divert patients to hotels instead of requiring continued hospital-based isolation, thus preserving critical capacity in our city’s largest public hospital. With continued COVID-19 surges and outbreaks among homeless individuals in congregate settings,^25^ ongoing support of hotel-based I/Q models is warranted.

Many individuals referred from ZSFG to I/Q hotels were ineligible owing to behavioral health needs that exceeded what could be provided in independent hotel rooms despite available addiction care telehealth consultation and regular wellness checks by behavioral health clinicians. Additional settings outside the hospital, beyond the hotel-based system of care,^26^ are needed to accommodate individuals with severe mental health and substance use disorders. Furthermore, jurisdictions should look beyond these emergency responses for this population and invest in affordable and permanent supportive housing programs.^27^

We also found that premature discontinuation of I/Q was common—19% of the cohort left before completing their prescribed stay. The odds of premature discontinuation of I/Q were greatest among unsheltered homeless and those offered I/Q for quarantine because of exposure (vs isolation due to infection). Poor adherence to self-isolation has been reported at higher rates elsewhere^28^ and is likely multifactorial. A review of the psychological impact of quarantine has suggested that lack of information about its purpose may be associated with perceived difficulties with adherence.^29^ Risk of premature discontinuation of I/Q may be compounded among those without symptoms who face prolonged indoor confinement, disruptions in their usual routine, and social and physical isolation. Mistrust of services has previously been associated with unsheltered homeless individuals’ hesitation to stay indoors or accept assistance.^30,31^ Increasing retention may require enhanced communication and trust building with clients and homeless services about the rationale and support for I/Q,^32^ as well as additional incentives, improved harm-reduction efforts, and other innovative solutions.^30,33,34^ Why premature discontinuation was more likely among female and younger guests is unclear; however, a qualitative study is currently under way to explore these associations and inform improvements to our current model of I/Q support and harm-reduction services.

### Limitations and Strengths

Our study had several limitations. First, we were unable to implement a comprehensive electronic record to systematically track the clinical progress and disposition of all guests across referral sources as we quickly launched the I/Q hotel–based system. However, our ability to integrate CCMS data for most individuals allowed us to capture critical information on the length of homelessness and jail health encounters. In addition, our ability to interrogate the ZSFG electronic medical record highlighted why individuals were ineligible for I/Q hotel stays. Second, while the trend in reduced hospital length of stay was encouraging, it may have reflected, in part, improved SARS-CoV-2 test turnaround time for persons under investigation during the study period. Our rapid launch of the I/Q program left insufficient time before implementation for a suitable counterfactual to explore how this system of care was associated with ZSFG length of hospital stay or hospital census among patients experiencing homelessness with confirmed or suspected COVID-19. Third, we were unable to differentiate between treated vs untreated mental health conditions that could potentially be associated with premature discontinuation of I/Q. Fourth, the full I/Q system of care developed in San Francisco may not be generalizable to all settings. However, as in other jurisdictions,^9^ we relied heavily on non–public health civil service workers to serve as impromptu hotel managers and support staff, revealing similar challenges in onboarding a large workforce with limited experience serving homeless populations and other marginalized communities.

## Conclusions

The COVID-19 pandemic has exacerbated preexisting structural inequities that place individuals experiencing homelessness and those living in congregate settings and dense households at high risk for infection. San Francisco rapidly scaled a hotel-based I/Q system of care that safely delivered medical and behavioral health support to more than 1000 individuals referred from various health care and community settings while helping to preserve hospital capacity. Community-informed strategies to improve retention and address behavioral health needs not met by the current I/Q hotel model are priorities as we face subsequent waves of infection.

## Notes

Supplement. **eTable 1.** Characteristics of Individuals Admitted to Isolation and Quarantine (I/Q) Hotels by Match to Administrative Records **eTable 2.** Factors Associated With Premature Discontinuation of Isolation and Quarantine (I/Q), Modeled With Interaction Term (N=954) **eTable 3.** Sensitivity Analysis Exploring Factors Associated With Premature Discontinuation of Isolation and Quarantine (I/Q), Limited to Patients With CCMS Administrative Data (N=857) **eMethods.** GetCare Online Isolation and Quarantine Hotel Referral Form
