# Supplementary material for: Assessment of a Hotel-Based COVID-19 Isolation and Quarantine Strategy for Persons Experiencing Homelessness
Source: JAMA Netw Open. 2021 Mar 2;4(3):e210490. doi: 10.1001/jamanetworkopen.2021.0490 (PMC7926291; doi:10.1001/jamanetworkopen.2021.0490)
Supplement: Supplement. — eTable 1. Characteristics of Individuals Admitted to Isolation and Quarantine (I/Q) Hotels by Match to Administrative Records eTable 2. Factors Associated With Premature Discontinuation of Isolation and Quarantine (I/Q), Modeled With Interaction Term (N=954) eTable 3. Sensitivity Analysis Exploring Factors Associated With Premature Discontinuation of Isolation and Quarantine (I/Q), Limited to Patients With CCMS Administrative Data (N=857) eMethods. GetCare Online Isolation and Quarantine Hotel Referral Form [file jamanetwopen-e210490-s001.pdf]

Supplementary Online Content

Fuchs JD, Carter HC, Evans J, et al. Assessment of a hotel-based COVID-19 isolation and quarantine strategy for persons experiencing homelessness. *JAMA Netw Open.* 2021;4(3):e210490. doi:10.1001/jamanetworkopen.2021.0490

eTable 1. Characteristics of Individuals Admitted to Isolation and Quarantine (I/Q) Hotels by Match to Administrative Records
eTable 2. Factors Associated With Premature Discontinuation of Isolation and Quarantine (I/Q), Modeled With Interaction Term (N=954)
eTable 3. Sensitivity Analysis Exploring Factors Associated With Premature Discontinuation of Isolation and Quarantine (I/Q), Limited to Patients With CCMS Administrative Data (N=857)
eMethods. GetCare Online Isolation and Quarantine Hotel Referral Form

This supplementary material has been provided by the authors to give readers additional information about their work.

© 2021 Fuchs JD et al. *JAMA Network Open.*

eTable 1. Characteristics of Individuals Admitted to Isolation and Quarantine (I/Q) Hotels by Match to Administrative Records

	Matched		Unmatched		
	**N**	**%**	**N**	**%**	*P* value
**Total**	907	89.9	102	10.1	
**Premature discontinuation of I/Q**					
No	697	81.3	79	80.6	0.86
Yes	160	18.7	19	19.4	
**Age**					
<40	324	35.7	72	70.6	<0.001
40-49	239	26.4	10	9.8	
50 - 59	206	22.7	10	9.8	
>=60	138	15.2	10	9.8	
**Gender**					
Male	689	76.0	67	65.7	0.01*
Female	208	22.9	31	30.4	
Transgender	6	0.7	4	3.9	
Other/Unknown	4	0.4	0	0.0	
**Race/Ethnicity**					
White	232	25.6	3	2.9	<0.001*
Black	164	18.1	23	22.5	
Latinx	405	44.7	49	48.0	
Asian/Pacific Islander	68	7.5	10	9.8	
Native American	8	0.9	5	4.9	
Multi-Ethnic	14	1.5	7	6.9	
Refused/Unknown	16	1.8	5	4.9	
**Living Situation**					
Home/Apartment/RV/Trailer	246	27.1	36	35.3	<0.001*
Sheltered Homeless	274	30.2	21	20.6	
Unsheltered Homeless	197	21.7	9	8.8	
Single Room Occupancy Hotel	123	13.6	10	9.8	
Congregate Living Setting	9	1.0	3	2.9	
Other/Unknown	58	6.4	23	22.5	
**Referral Source**					
Outpatient	89	9.8	12	11.8	<0.001
Hospital	336	37.0	31	30.4	
Homeless Service Provider/Shelter	160	17.6	9	8.8	
Surveillance/Field Testing	96	10.6	10	9.8	
Other	141	15.5	12	11.8	

© 2021 Fuchs JD et al. *JAMA Network Open.*

Missing	85	9.4	28	27.5	
**COVID Status**					
COVID-19 Diagnosis	420	46.3	43	42.2	0.20*
PUI	344	37.9	35	34.3	
Close Contact	124	13.7	22	21.6	
None of the above	4	0.4	1	1.0	
Missing	15	1.7	1	1.0	

*Fisher's exact *P*-value

Compared to individuals who were not matched to the CCMS database, matched individuals were significantly older (median [IQR]: 45 years [35-55] vs. 27 [13-44], p<0.01), more likely to be referred from a hospital or homeless service provider (37% vs. 30% and 18% vs. 9% respectively, p<0.01), to be homeless (52% vs. 29%, p<0.01), more likely to be White compared to other race/ethnicities (26% vs. 3%, p<0.01), and more likely to be male (76% vs. 66%, p<0.01). There were no differences in COVID status or in the proportion who prematurely discontinued isolation and quarantine.

Abbreviations: RV- Recreational Vehicle; PUI- Person Under Investigation

© 2021 Fuchs JD et al. *JAMA Network Open.*

eTable 2. Factors Associated With Premature Discontinuation of Isolation and Quarantine (I/Q), Modeled With Interaction Term (N=954)

Factor	AOR (95% CI), *P* value[1]
**Age, years**	
>=60	1 [Reference]
50-59	1.8 (0.9 - 3.6), .08
40-49	1.8 (0.9 - 3.5), .09
<40	2.6 (1.4 - 4.9), .004
**Gender**	
Male	1 [Reference]
Female	1.8 (1.2 - 2.7), .006
Transgender/Other	0.4 (0.1 - 2.2), .30
**Race/Ethnicity**	
Non-Black	1 [Reference]
Black	1.5 (0.3 - 7.4), .65
**Living Situation**	
Home/Apartment/RV/Trailer	1 [Reference]
Sheltered Homeless	1.4 (0.7 - 2.8), .37
Unsheltered Homeless	5.0 (2.6 - 9.5), <.001
Congregate Living Setting/SRO	1.5 (0.7 - 3.1), .26
Other/Unknown	1.2 (0.5 - 3.1), .63
**Race/Ethnicity (Black) * Living Situation[2]**	
Race/Ethnicity (Black) * Home/Apartment/RV/Trailer[3]	–
Race/Ethnicity (Black) * Sheltered Homeless	2.1 (0.4 - 12.2), .41
Race/Ethnicity (Black) * Unsheltered Homeless	0.9 (0.2 - 5.3), .91
Race/Ethnicity (Black) * Congregate Living Setting/SRO	0.3 (0.02 - 4.9), .42
Race/Ethnicity (Black) * Other/Unknown	1 [Reference]
**Referral Source**	
Outpatient	1 [Reference]
Hospital	1.1 (0.6 - 1.9), .86
Homeless Service Provider/Shelter	0.5 (0.2 - 1.1), .07
Surveillance/Field Testing	0.7 (0.3 - 1.6), .37
Other	0.6 (0.3 - 1.2), .14
Missing	0.5 (0.2 - 1.1), .09
**COVID Status**	
COVID-19 Diagnosis	1 [Reference]
PUI	1.5 (0.9 - 2.5), .08
Close Contact	2.7 (1.5 - 4.6), .001
None of the Above	3.6 (0.5 - 25.5), .20

© 2021 Fuchs JD et al. *JAMA Network Open.*

Missing	5.0 (1.0 - 23.9), .05
**Elixhauser medical condition**	
No	1 [Reference]
Yes	1.4 (0.9 - 2.3), .14
**Elixhauser mental health disorder**	
No	1 [Reference]
Yes	0.9 (0.6 - 1.5), .76
**Elixhauser substance use disorder**	
No	1 [Reference]
Yes	0.9 (0.5 - 1.5), .56
**Jail stay in past year**	
No	1 [Reference]
Yes	1.2 (0.7 - 2.2), .49

[1] Model adjusted for calendar week of referral
[2] *P*-value for interaction = 0.20
[3] Category omitted due to zero cell

Abbreviations: AOR: Adjusted Odds Ratio; CI: Confidence Interval; RV- Recreational Vehicle; SRO- Single Room Occupancy Hotel; PUI- Person Under Investigation

© 2021 Fuchs JD et al. *JAMA Network Open.*

eTable 3. Sensitivity Analysis Exploring Factors Associated With Premature Discontinuation of Isolation and Quarantine (I/Q), Limited to Patients With CCMS Administrative Data (N=857)

**Factor**	**AOR (95% CI), *P* value^**
**Age, years**	
>=60	1 [Reference]
50-59	2.5 (1.1 - 5.2), .02
40-49	2.5 (1.2 - 5.2), .02
<40	3.9 (1.8 - 8.2), <.001
**Gender**	
Male	1 [Reference]
Female	1.9 (1.2 - 3.0), .003
Transgender/Other	0.2 (0.01 - 1.8), .13
**Race/Ethnicity**	
White	1 [Reference]
Black	1.7 (1.0 - 2.9), .07
Latinx	0.9 (0.5 - 1.6), .80
Asian/Pacific Islander	0.6 (0.2 - 1.6), .32
Multi-Ethnic/Other/Unknown	1.4 (0.6 - 3.5), .48
**Living Situation**	
Home/Apartment/RV/Trailer	1 [Reference]
Sheltered Homeless	1.4 (0.7 - 2.9), .35
Unsheltered Homeless	3.7 (1.9 - 7.4), <.001
Congregate Living Setting/SRO	1.2 (0.5 - 2.5), .67
Other/Unknown	1.0 (0.3 - 3.3), .94
**Referral Source**	
Outpatient	1 [Reference]
Hospital	1.2 (0.6 - 2.4), .62
Homeless Service Provider/Shelter	0.5 (0.2 - 1.2), .13
Surveillance/Field Testing	0.7 (0.3 - 1.7), .41
Other	0.6 (0.3 - 1.2), .14
Missing	0.4 (0.2 - 1.1), .07
**COVID Status**	
COVID-19 Diagnosis	1 [Reference]
PUI	1.5 (0.9 - 2.6), .10
Close Contact	2.7 (1.5 - 5.0), .001
None of the above	9.0 (1.0 - 85.1), .05
Missing	3.7 (0.6 - 21.2), .14
**Elixhauser medical condition**	

© 2021 Fuchs JD et al. *JAMA Network Open.*

No	1 [Reference]
Yes	1.4 (0.9 - 2.2), .20
**Elixhauser mental health disorder**	
No	1 [Reference]
Yes	0.9 (0.6 - 1.6), .80
**Elixhauser substance use disorder**	
No	1 [Reference]
Yes	0.8 (0.5 - 1.4), .41
**Jail stay in past year**	
No	1 [Reference]
Yes	1.2 (0.7 - 2.2), .50
**Years homeless, per year**	1.0 (1.0 – 1.1), .18

^ Model adjusted for calendar week of referral.

Abbreviations: AOR: Adjusted Odds Ratio; CI: Confidence Interval; RV- Recreational Vehicle; SRO- Single Room Occupancy Hotel; PUI- Person Under Investigation

© 2021 Fuchs JD et al. *JAMA Network Open.*

eMethods. GetCare Online Isolation and Quarantine Hotel Referral Form

San Francisco
Health Network

SAN FRANCISCO DEPARTMENT OF PUBLIC HEALTH

The City and County of San Francisco has secured hotel rooms and other spaces to help individuals exposed to or living with COVID-19 isolate themselves and prevent transmission to others. Please complete the following intake form to determine if these rooms and spaces can meet the needs of your clients and patients.

If you are having difficulties using or submitting this form, please contact our Intake Hotline at 628-652-2820.

Referring Party Information:

I. WHO ARE YOU?

STAFF NAME:

First Name: *

Last Name: *

Staff Role (Case Manager, Physician, etc.): *

Program Type Category: *

DIRECT CONTACT INFO: *

Phone Type:

Phone:

Email Type:

Email:

Which method should we use to contact you?: *

Demographic Information on the Person Being Referred:

II. WHO ARE YOU REFERRING?

Client First Name: *

Client Middle Name:

Client Last Name: *

Nickname/Alias:

Date of Birth: *

Full Social Security Number:

Client Cell Phone Number: *

☐ If the client doesn't have access to a cell phone, describe how client can be contacted

Preferred contact method:

Preferred Language: *

Sexual orientation/gender identity, race/ethnicity, living situation, and medical record number:

What is the client's gender? (select one that best describes their current gender identity.) *

Preferred Gender Pronouns:

What was the client's sex at birth?:

How does the client describe their sexual orientation or sexual identity?

What is the client's race/ethnicity?: *

MRN (San Francisco Health Network):

MRN (Other):

How would the client describe their living situation?:*

Symptom and Testing Information:

III. ARE THEY UNWELL?

Tell us about your client's symptoms. *

☐ Fever (above 100.4 F) ☐ Cough ☐ Fatigue ☐ Malaise ☐ GI ☐ Sore Throat ☐ Shortness of Breath ☐ None

☐ Other, Please describe:

If no current fever, any fever in last 7 days?:

Date of first symptoms: *

COVID Status: *

○ COVID-19 Positive (person has tested positive for COVID-19)

○ Asymptomatic Confirmed Contact (ACC; person has confirmed contact with a COVID-19 positive individual and has no current symptoms)

○ Person Under Investigation (PUI): symptomatic patient needing testing or awaiting test results; do NOT select if patient is asymptomatic

○ None of the Above

Needs Testing ○ Yes ○ No ○ Unsure

Date of Test:

Location of Test:

☐ Is this a testing event?

Test Outcome

Test Outcome Date

Remove −

Add +

Relevant Medical History/Co-Morbidities:

Relevant Medical History:

Does your client have any pre-existing medical conditions? Check all that apply:

☐ Chronic lung disease (asthma/emphysema/COPD)
☐ Chronic renal disease
☐ Current smoker
☐ Severe Obesity (BMI >40)
☐ Other chronic disease:
☐ Diabetes Mellitus
☐ Chronic liver disease
☐ Former smoker
☐ Hypertension
☐ Cardiovascular disease, including congestive heart failure
☐ Immunocompromised (cancer, HIV, bone marrow or organ transplant, or other immune weakening conditions)
☐ Neurological/neurodevelopmental

Pregnant ○ Yes ○ No ○ Unk/NA

Does the patient have any medication allergies? *

Current Medications:

How many days' supply do they have?:

Remove −

Add +

Reason they need help isolating:

IV. DO THEY NEED US FOR PLACEMENT IN AN ISOLATION AND QUARANTINE SITE? *

○ Person experiencing sheltered homeless (inhabiting shelter, Shelter In Place hotel site, etc.)

○ Person experiencing unsheltered homelessness (encampment, tent, etc.)

○ Person living in a shared space and unable to stay >6 feet away from others

○ Person sharing a bathroom and person unable to maintain appropriate hygiene (wash hands, wipe down surfaces after use, etc.)

○ None of the above apply.

© 2021 Fuchs JD et al. *JAMA Network Open.*

Making sure they can manage safely in the hotel room:

V. CAN THEY MANAGE AT A CITY SPONSORED COVID-19 CONTAINMENT SITE?

Hotel rooms are for clinically and behaviorally stable patients who can answer daily questions about their symptoms.

Is the client able to care for their own hygiene, toileting, medication management and check their own temperature? A Homebridge/IHSS referral may be coordinated for the patient if assistance is needed in some IALDs/ALDs. *

Has the client demonstrated any impulse or behavioral control issues during a recent hospital stay or participation in a program? *

Does the client have symptoms that may need acute medical care or hospitalization? *

Please inform the patient that they will be staying in a hotel room or congregate setting for up to 7-14 days. They will be expected to stay inside the room or at the congregate site the entire time. Meals will be delivered three times a day, but they cannot order room service, etc. Nurses and other support staff will be available to assist with medical needs and questions. Has the client agreed to stay in a City sponsored room or congregate site for the remainder of their care? *

Do you think client would do better at or would prefer a congregate setting, with more ability to go outside and interact with others, over an individual hotel room? *

Determining the best site placement:

VI. HELP US PLACE THEM IN THE RIGHT PLACE

Does the client have any ADA needs? For example, do they need a wheelchair accessible building? *

Will the client bring a partner or a family member with them? *

Does the client have a companion animal that they would need to bring with them? *

Does the client have a bicycle that they would need to bring with them? *

Providing support while they're in the hotel:

VII. HELP US SUPPORT THEM WHILE IN OUR CARE

Does the client have any chronic medical needs that would require Nursing support? *

Does the client use any drugs, including alcohol or cigarettes, regularly? *

Does the client take methadone/suboxone? *

Does the patient receive mental health services? *

Does the patient take any psychiatric medication? *

Does the patient have a serious mental health diagnosis? *

Does the patient receive Intensive Case Management (ICM) services? *

Does the patient have any food sensitivities or dietary restrictions?

© 2021 Fuchs JD et al. *JAMA Network Open.*

Discharge Plan and Notes:

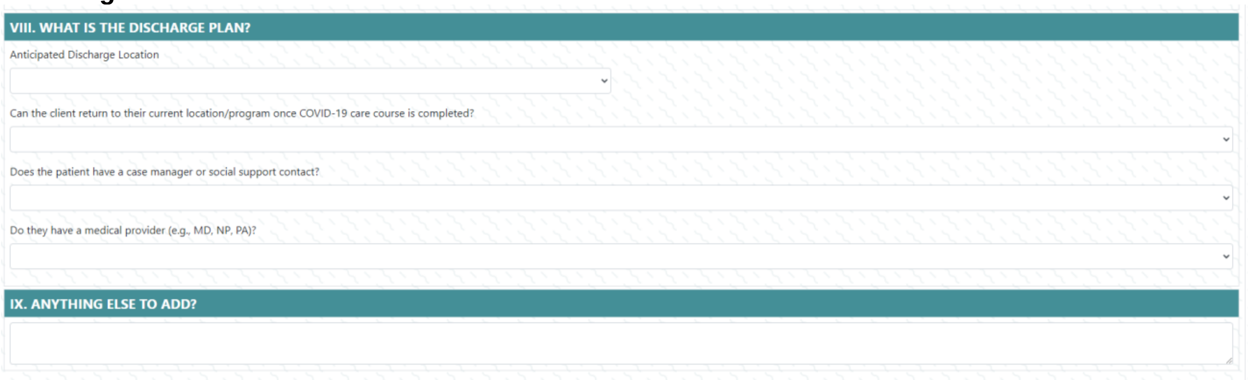
VIII. WHAT IS THE DISCHARGE PLAN?

Anticipated Discharge Location

Can the client return to their current location/program once COVID-19 care course is completed?

Does the patient have a case manager or social support contact?

Do they have a medical provider (e.g., MD, NP, PA)?

IX. ANYTHING ELSE TO ADD?

© 2021 Fuchs JD et al. *JAMA Network Open.*
